# Inhibition of Vesicular Glutamate Transporters (VGLUTs) with Chicago Sky Blue 6B Before Focal Cerebral Ischemia Offers Neuroprotection

**DOI:** 10.1007/s12035-023-03259-1

**Published:** 2023-02-18

**Authors:** Bartosz Pomierny, Weronika Krzyżanowska, Alicja Skórkowska, Jakub Jurczyk, Beata Bystrowska, Bogusława Budziszewska, Joanna Pera

**Affiliations:** 1 Laboratory for Stroke Research, Department of Toxicological Biochemistry, Faculty of Pharmacy, Jagiellonian University Medical College, Medyczna 9, 30-688 Kraków, Poland; 2 Department of Neurology, Faculty of Medicine, Jagiellonian University Medical College, Botaniczna 3, 31-503 Kraków, Poland

**Keywords:** Brain ischemia, Vesicular glutamate transporters, Excitotoxicity, Middle cerebral artery occlusion, Brain preconditioning

## Abstract

Brain ischemia is one of the leading causes of death and long-term disability in the world. Interruption of the blood supply to the brain is a direct stimulus for many pathological events. The massive vesicular release of glutamate (Glu) after ischemia onset induces excitotoxicity, which is a potent stress on neurons. Loading of presynaptic vesicles with Glu is the first step of glutamatergic neurotransmission. Vesicular glutamate transporters 1, 2, and 3 (VGLUT1, 2, and 3) are the main players involved in filling presynaptic vesicles with Glu. VGLUT1 and VGLUT2 are expressed mainly in glutamatergic neurons. Therefore, the possibility of pharmacological modulation to prevent ischemia-related brain damage is attractive. In this study, we aimed to determine the effect of focal cerebral ischemia on the spatiotemporal expression of VGLUT1 and VGLUT2 in rats. Next, we investigated the influence of VGLUT inhibition with Chicago Sky Blue 6B (CSB6B) on Glu release and stroke outcome. The effect of CSB6B pretreatment on infarct volume and neurological deficit was compared with a reference model of ischemic preconditioning. The results of this study indicate that ischemia upregulated the expression of VGLUT1 in the cerebral cortex and in the dorsal striatum 3 days after ischemia onset. The expression of VGLUT2 was elevated in the dorsal striatum and in the cerebral cortex 24 h and 3 days after ischemia, respectively. Microdialysis revealed that pretreatment with CSB6B significantly reduced the extracellular Glu concentration. Altogether, this study shows that inhibition of VGLUTs might be a promising therapeutic strategy for the future.

## Introduction

Stroke is one of the major causes of death and disability in adults worldwide. Approximately 80% of strokes are ischemic strokes caused by cessation or severe impairment of cerebral blood flow [1, 2]. Unfortunately, there is no effective treatment applicable for all stroke patients, and the reperfusion therapy can be used in only a small proportion of patients. In addition, there are no neuroprotective strategies that could be used in stroke.

Glutamate (Glu) is the predominant excitatory neurotransmitter in the mammalian brain. During the early phase of cerebral ischemia, there is a large increase in extracellular Glu, which causes the overstimulation of glutamatergic receptors and leads to irreversible damage to nerve and glial cells [3]. Regulatory mechanisms in ischemic conditions are insufficient to control Glu levels. Previous attempts at reducing excitotoxicity through inhibition of the N-methyl-D-aspartate receptor (NMDAR) or stimulation of Glu astrocytic uptake (e.g., excitatory amino acid transporters 1 and 2) failed in clinical trials. However, these studies were focused on targets of already released Glu. There is little data about the efficacy of the presynaptic modulation of Glu release in cerebral ischemia. Vesicular glutamate transporters (VGLUTs), which belong to the type I phosphate transporter family (SLC17 family), are the only group known to be responsible for Glu loading into vesicles in presynaptic terminals. Three VGLUT isoforms have been identified: VGLUT1 (SLC17A7), VGLUT2 (SLC17A6), and VGLUT3 (SLC17A8). VGLUT 1 and VGLUT2 are extensively expressed throughout the brain, mainly on glutamatergic neurons, but their distribution differs. These isoforms play a vital role in glutamatergic transmission. VGLUT1 or VGLUT2 knockout mice die just after weaning or after birth, respectively. The expression of VGLUT3 is limited to nonglutamatergic neurons in the dorsal and ventral striatum, cortex, and hippocampus [4, 5]. The expression levels of VGLUTs determine the amount of Glu loaded into presynaptic vesicles and its subsequent release into the synaptic cleft; thus, excitotoxicity is directly related with the level and activity of VGLUT1 and VGLUT2 [6, 7]. These transporters are implicated in psychiatric and neurological diseases in which excitotoxicity is the component of their pathomechanism, including schizophrenia [8, 9], brain ischemia [10], Alzheimer’s disease [11, 12], and Parkinson’s disease [13–15]. Thus, a therapeutic strategy aimed at the inhibition of VGLUTs could have exert beneficial effect in the treatment of mentioned diseases. Since there are no VGLUT inhibitors with appropriate specificity or drug-likeness, this study is to verify the role of VGLUT1 and VGLUT2 as a therapeutical target in brain ischemia, and thus to intensify research on innovative compound of clinical relevancy.

In the present study, we aimed to characterize the influence of transient focal brain ischemia on the expression of VGLUT1 and VGLUT2 in rats. Next, we investigated how the inhibition of VGLUTs with Chicago Sky Blue 6B (CSB6B) pretreatment affects Glu release in the ischemic brain. CSB6B is a potent, however, nonselective inhibitor of VGLUTs [16–18]. We compared the effects of VGLUT inhibition on the severity of neurological deficits and the infarct volume with ischemic preconditioning (IP) as a reference method.

## Results

### Inhibition of VGLUTs Prior to MCAO Reduced the Neurological Deficit and Infarct Volume

**Fig. 1:**
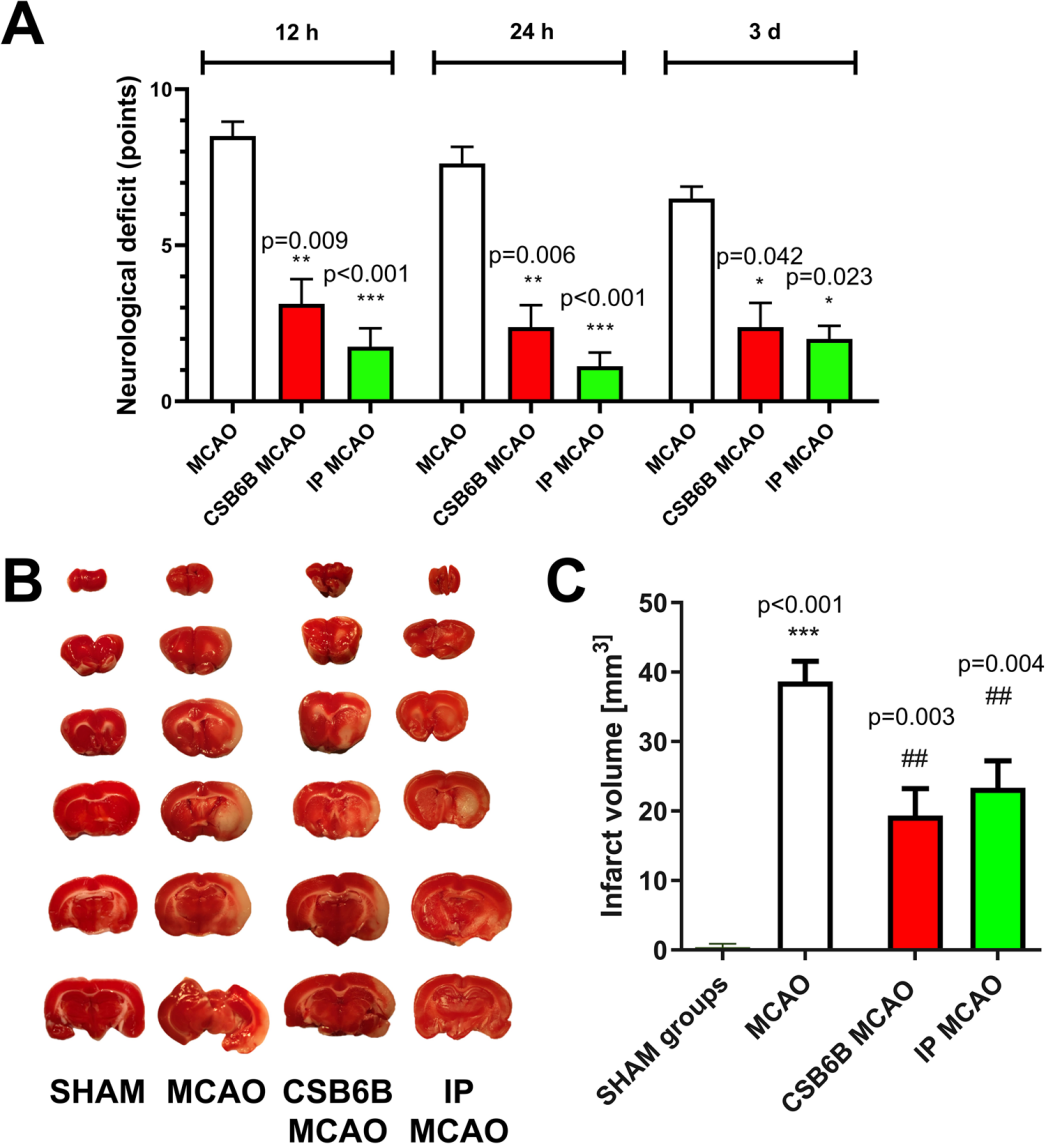
Role of ischemic preconditioning and CSB6B pretreatment on ischemic brain damage and its effect, the neurological deficit. **A** Neurological deficits were scored as described in “Methods” at 12 h, 24 h, and 3 d after reperfusion. There were no neurological deficits found in sham-operated animals. The difference between ischemic groups was determined by a Mann–Whitney *U* test (*n* = 8), the statistical significance assessed at *p* < 0.05. **B** Representative TTC staining of coronal (2 mm) brain sections, 24 h after reperfusion or sham procedure. Pale areas indicate the infarct. **C** The infarct volume of ipsilateral brain hemisphere corrected for oedema. The infarct volume in mm^3^ is shown as the mean ± SEM (*n* = 6). The difference was assessed using unpaired *t* test. The statistical significance was assessed at *p* < 0.05. For the infarct volume, a statistically significant difference between SHAM and MCAO groups was marked with *, whereas for comparison between ischemic groups with #. */# *p* < 0.05; **/## *p* < 0.01; ***/### *p* < 0.001

Pretreatment with CSB6B resulted in a significant reduction in neurological deficits compared to MCAO at each timepoint assessed (neurological deficit score, MCAO vs CSB6B MCAO: 8.50 ± 0.54 vs 3.125 ± 0.79, *p* < 0.01 for 12 h; 7.625 ± 0.49 vs 2.625 ± 0.86, *p* < 0.01 for 24 h; 6.50 ± 0.58 vs 3.0 ± 0.93, *p* < 0.05 for 3 d after reperfusion; Fig. 1A). This effect was similar to that seen after IP (scores for IP MCAO: 2.5 ± 1.18, *p* < 0.001 for 12 h; 1.125 ± 0.44, *p* < 0.001 for 24 h; 2.0 ± 0.43, *p* < 0.05 for 3 d). Additionally, the infarct volume was significantly smaller in animals pretreated with CSB6B or subjected to IP than in the MCAO-only group (38.65 ± 2.898 mm^3^, MCAO vs 16.13 ± 6.25 mm^3^, *p* < 0.001, CSB6B MCAO vs 23.34 ± 3.87 mm^3^, *p* < 0.01, IP MCAO; Fig. 1B, C).

### The Effect of Cerebral Ischemia and CSB6B on the mRNA and Protein Expression of VGLUT1 and VGLUT2

**Fig. 2:**
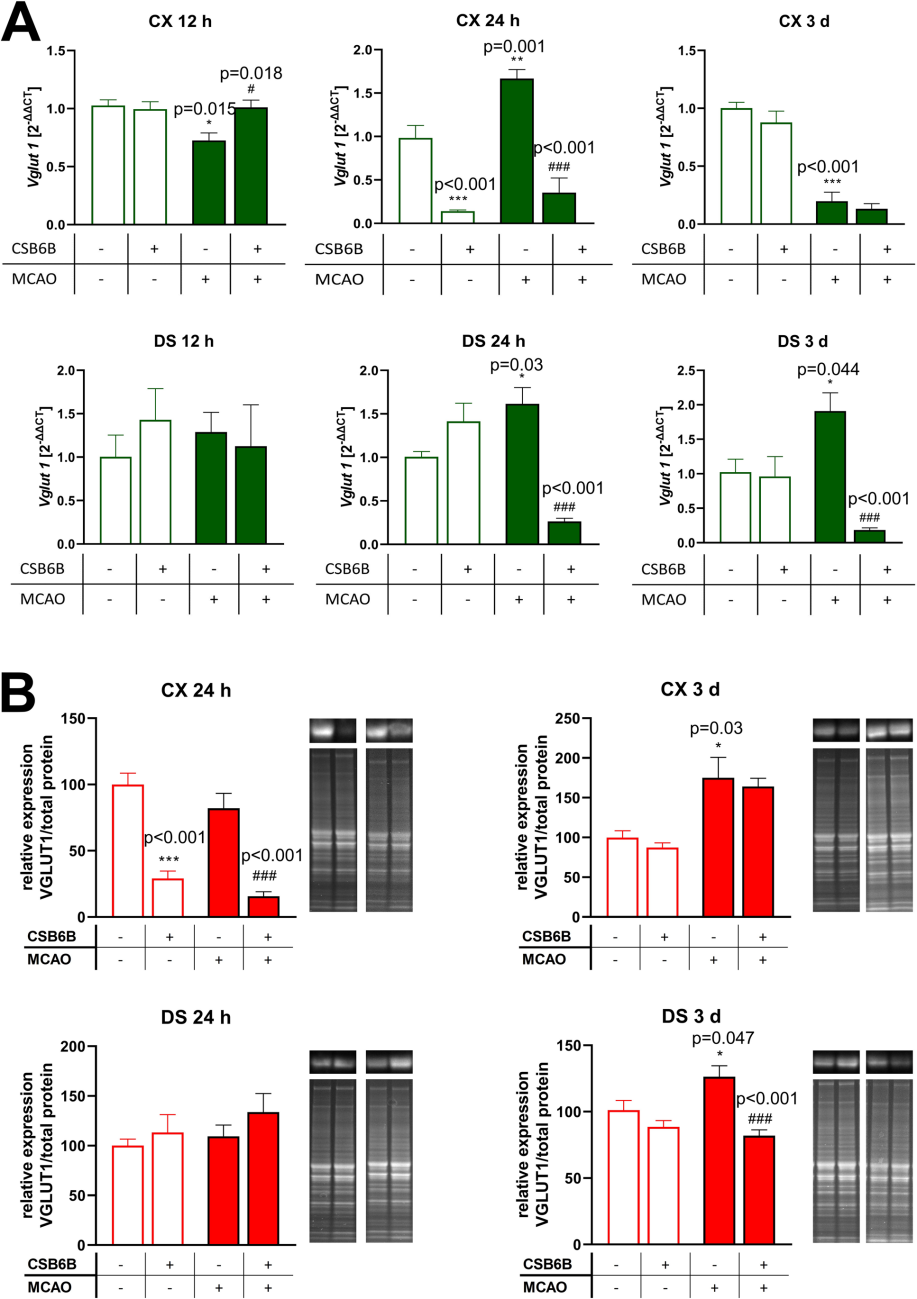
The effect of MCAO and CSB6B pretreatment on the expression of VGLUT1 mRNA (**A**) and protein (**B**) in the spatiotemporal context. **A** Results of RT-qPCR analysis of *Vglut1* are expressed as the mean fold change of *Vglut1* ± SEM (*n* = 6). Fold change was calculated using 2^−ΔΔCt^ method and three reference genes, *Ppia*, *Ywhaz*, and *Hprt*. Results for each experimental group are related to SHAM group. **B** Western blot results of VGLUT1 protein expression were normalized against SHAM group for which the expression is equal to 100% and are expressed as the means ± SEM (*n* = 6). On the right of each graph, representative immunoblots are presented with reference to the total protein concentration. Due to large number of experimental groups, ischemic groups and sham-operated groups were analyzed on separate gels, however, at the same run, the same experimental conditions and moreover, an additional gel with MCAO and SHAM groups was analyzed. mRNA and protein data were analyzed using one-way ANOVA, followed by Sidak *post ho*c test (*p < 0.05, **p < 0.01, ***p < 0.001 for comparisons vs SHAM; #*p* < 0.05, ##*p* < 0.01 for comparisons vs MCAO)

Twelve hours after MCAO, a significant reduction in VGLUT1 mRNA was observed in the frontal cortex but not in the dorsal striatum when compared to the SHAM group (Fig. 2A). In animals subjected to MCAO and pretreated with CSB6B, the VGLUT1 mRNA levels in both investigated areas were like those in control rats. Similarly, CSB6B administration alone did not alter VGLUT1 mRNA expression. In contrast, 24 h after reperfusion, a significant increase in VGLUT1 mRNA levels was observed in both the frontal cortex and the dorsal striatum, whereas the application of CSB6B before MCAO significantly reduced the levels of VGLUT1 mRNA in both brain areas. Additionally, the sole injection of CSB6B reduced VGLUT1 mRNA expression in the frontal cortex. Three days after MCAO, VGLUT1 mRNA levels were significantly decreased in the frontal cortex but increased in the dorsal striatum when compared to SHAM animals. Pretreatment with CSB6B did not affect VGLUT1 mRNA levels in the frontal cortex but markedly reduced its expression in the dorsal stratum compared with MCAO. CSB6B itself did not alter VGLUT1 mRNA expression (Fig. 2A).

**Fig. 3:**
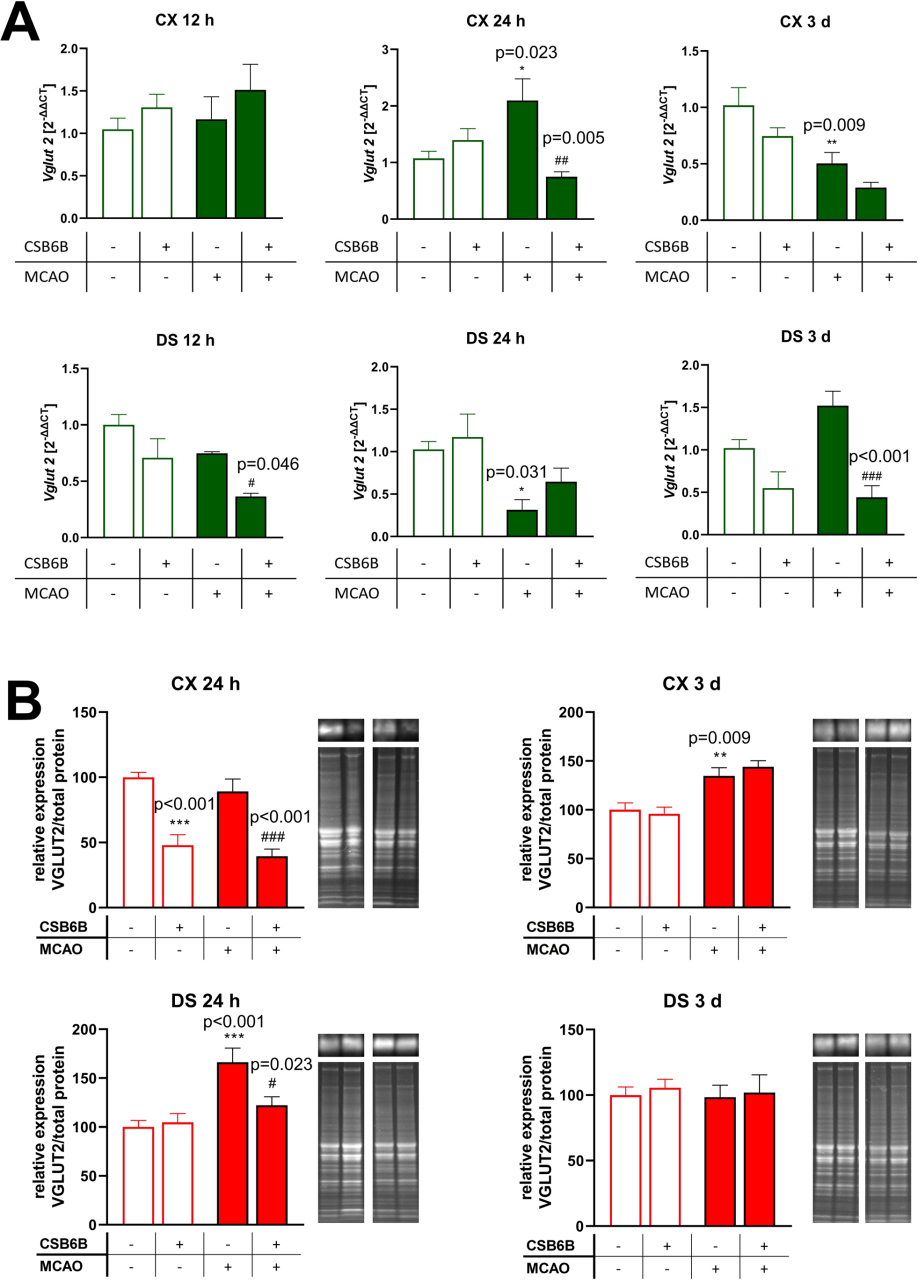
The effect of MCAO and CSB6B pretreatment on the expression of VGLUT2 mRNA (**A**) and protein (**B**) in the spatiotemporal context. **A** Results of RT-qPCR analysis of *Vglut2* are expressed as the mean fold change of *Vglut2* ± SEM (*n* = 6). Fold-change was calculated using 2^−ΔΔCt^ method and three reference genes, *Ppia*, *Ywhaz*, and *Hprt*. Results for each experimental group are related to SHAM group. **B** Western blot results of VGLUT2 protein expression were normalized against SHAM group for which the expression is equal to 100% and are expressed as the means ± SEM (*n* = 6). On the right of each graph, representative immunoblots are presented with reference to the total protein concentration. Due to large number of experimental groups, ischemic groups and sham-operated groups were analyzed on separate gels, however, at the same run, the same experimental conditions and moreover, an additional gel with MCAO and SHAM groups was analyzed. mRNA and protein data were analyzed using one-way ANOVA, followed by Sidak post hoc test (*p < 0.05, **p < 0.01, ***p < 0.001 for comparisons vs SHAM; #*p* < 0.05, ##*p* < 0.01 for comparisons vs MCAO)

VGLUT2 mRNA levels remained unchanged at 12 h after MCAO with or without CSB6B pretreatment or CSB6B itself in the frontal cortex (Fig. 3A). In the dorsal striatum, only MCAO preceded by CSB6B administration reduced the expression of VGLUT2 mRNA. Twenty-four hours after MCAO, VGLUT2 mRNA levels were increased in the frontal cortex but decreased in the dorsal striatum. CSB6B administration prior to MCAO significantly reduced VGLUT2 mRNA expression in the frontal cortex. In the dorsal striatum, no significant changes were observed. Three days after MCAO, VGLUT2 mRNA expression was in the opposite direction with downregulation in the frontal cortex but with no significant effect in the dorsal striatum. Pretreatment with CSB6B resulted in a significant reduction in VGLUT2 mRNA levels in the dorsal striatum but not in the frontal cortex in animals subjected to ischemia. There was no effect of CSB6B administration alone (Fig. 3A).

Next, we analyzed the expression of VGLUT1 and VGLUT2 at the protein level. When compared to control animals, VGLUT1 expression was significantly increased in both investigated structures 3 d after reperfusion (Fig. 2B). Administration of CSB6B resulted in a marked reduction in VGLUT1 protein levels in the frontal cortex in rats both subjected and not subjected to MCAO at 24 h. In the dorsal striatum, CSB6B reduced VGLUT1 expression in animals subjected to ischemia at 3 d.

Brain ischemia caused a significant increase in VGLUT2 expression in the dorsal striatum 24 h later, and this effect was not influenced by CSB6B pretreatment (Fig. 3B). In the frontal cortex, CSB6B administration resulted in a decrease in VGLUT2 levels in both ischemic and nonischemic rats at 24 h.

**Fig. 4:**
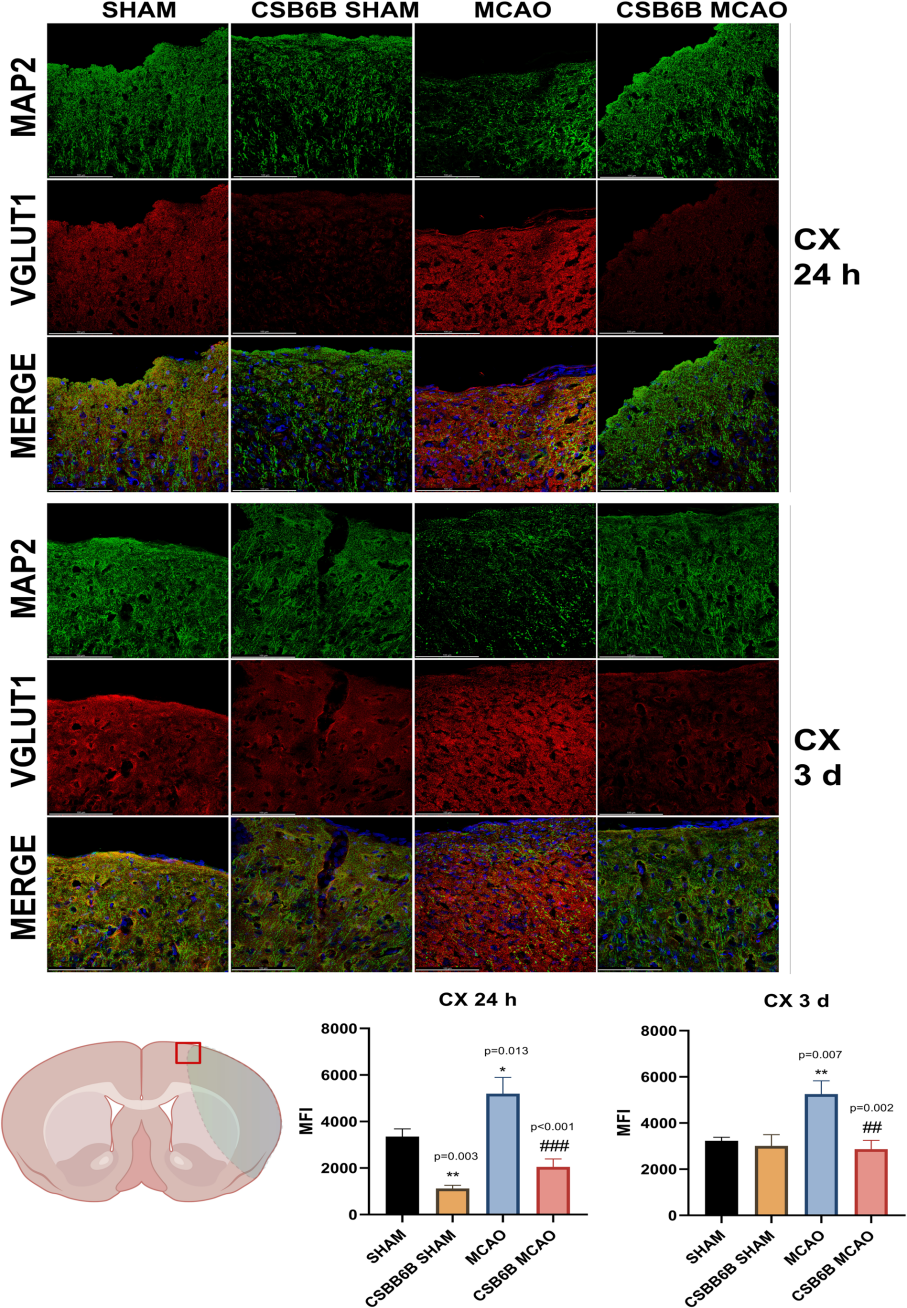
Expression of VGLUT1 in the cerebral cortex at 24 h and at 3 d after reperfusion. A total of 20 μm thick brain sections were double stained for MAP2 (green) and VGLUT1 (red). For each experimental group, *n* = 6 animals. Below on the left, on a brain diagram, the periinfarct area marked with red square indicates the location of performed analysis. Below on the right, the quantitative analysis of VGLUT1 mean fluorescence intensity (MFI). Data were analyzed with one-way ANOVA, followed by Sidak post hoc test (*p < 0.05, **p < 0.01 for comparisons vs SHAM; #*p* < 0.05, ##*p* < 0.01, ###*p* < 0.001 for comparisons vs MCAO). Scale bar = 100 µm

**Fig. 5:**
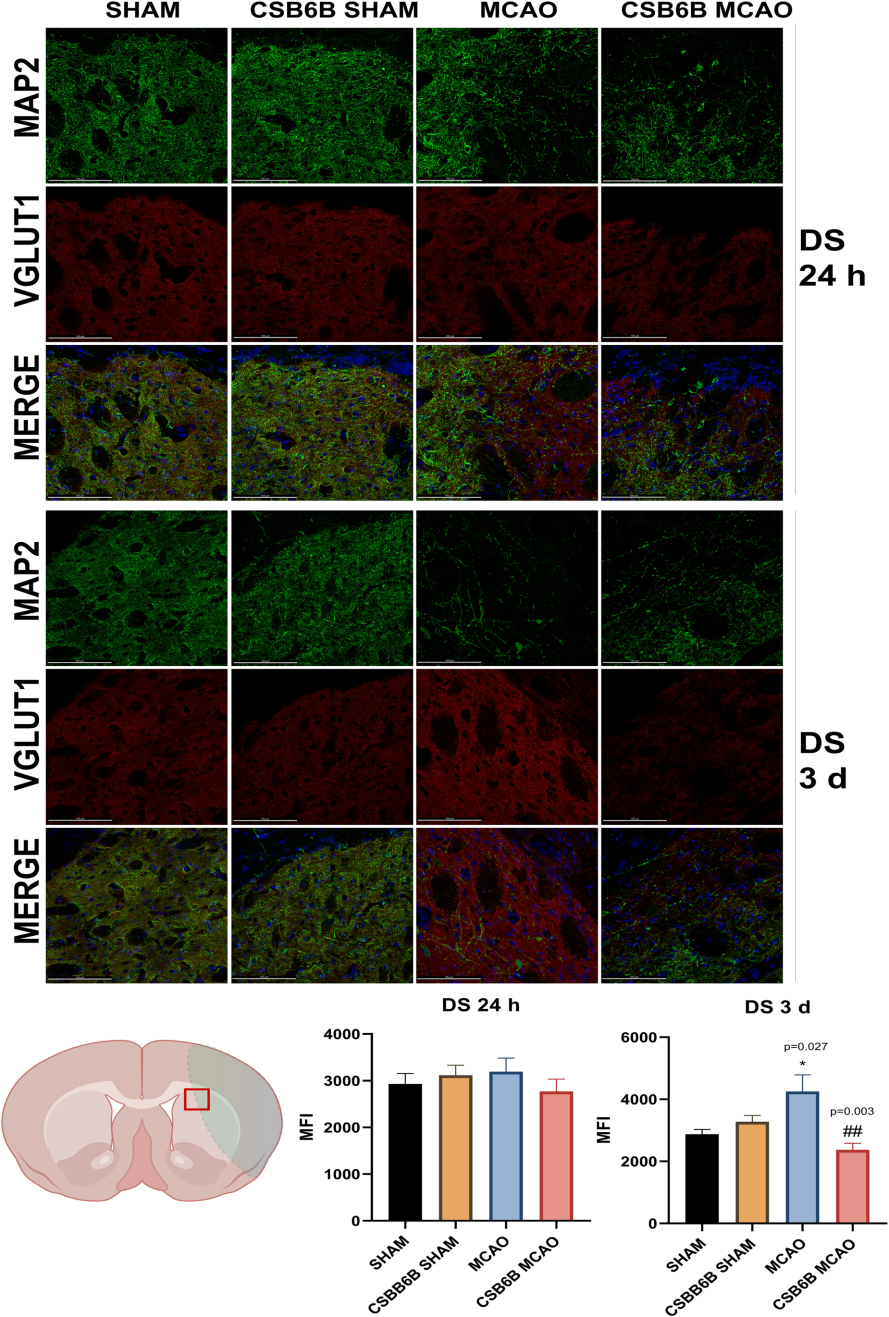
Expression of VGLUT1 in the dorsal striatum at 24 h and at 3 d after reperfusion. A total of 20 μm thick brain sections were double stained for MAP2 (green) and VGLUT1 (red). For each experimental group, *n* = 6 animals. Below on the left, on a brain diagram, the periinfarct area marked with red square indicates the location of performed analysis. Below on the right, the quantitative analysis of VGLUT1 mean fluorescence intensity (MFI). Data were analyzed with one-way ANOVA, followed by Sidak post hoc test (*p < 0.05 for comparisons vs SHAM; #*p* < 0.05, ##*p* < 0.01 for comparisons vs MCAO). Scale bar = 100 µm

**Fig. 6:**
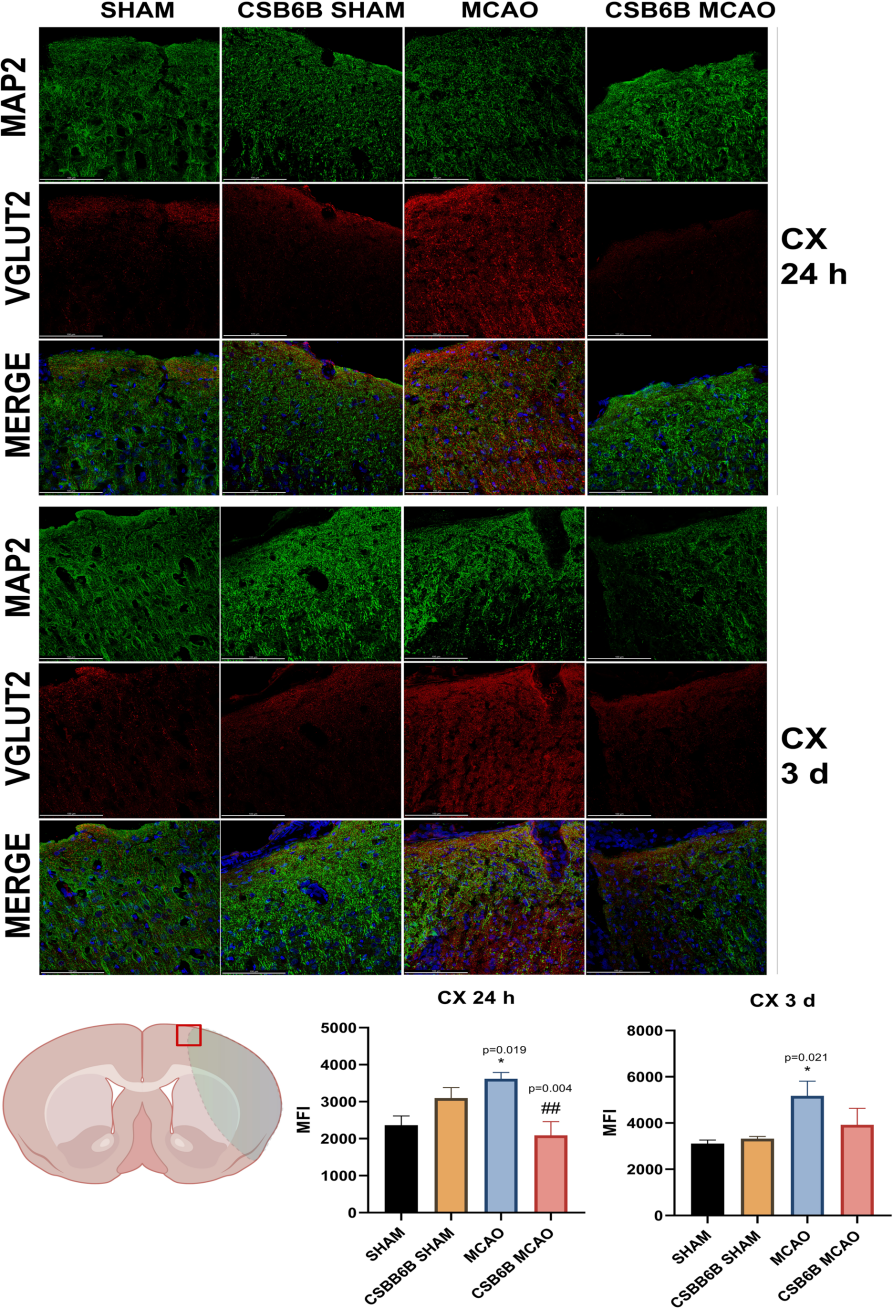
Expression of VGLUT2 in the cerebral cortex at 24 h and at 3 d after reperfusion. A total of 20 μm thick brain sections were double stained for MAP2 (green) and VGLUT2 (red). For each experimental group, *n* = 6 animals. Below on the left, on a brain diagram, the periinfarct area marked with red square indicates the location of performed analysis. Below on the right, the quantitative analysis of VGLUT2 mean fluorescence intensity (MFI). Data were analyzed with one-way ANOVA, followed by Sidak post hoc test (*p < 0.05 for comparisons vs SHAM; #*p* < 0.05, ##*p* < 0.01 for comparisons vs MCAO). Scale bar = 100 µm

**Fig. 7:**
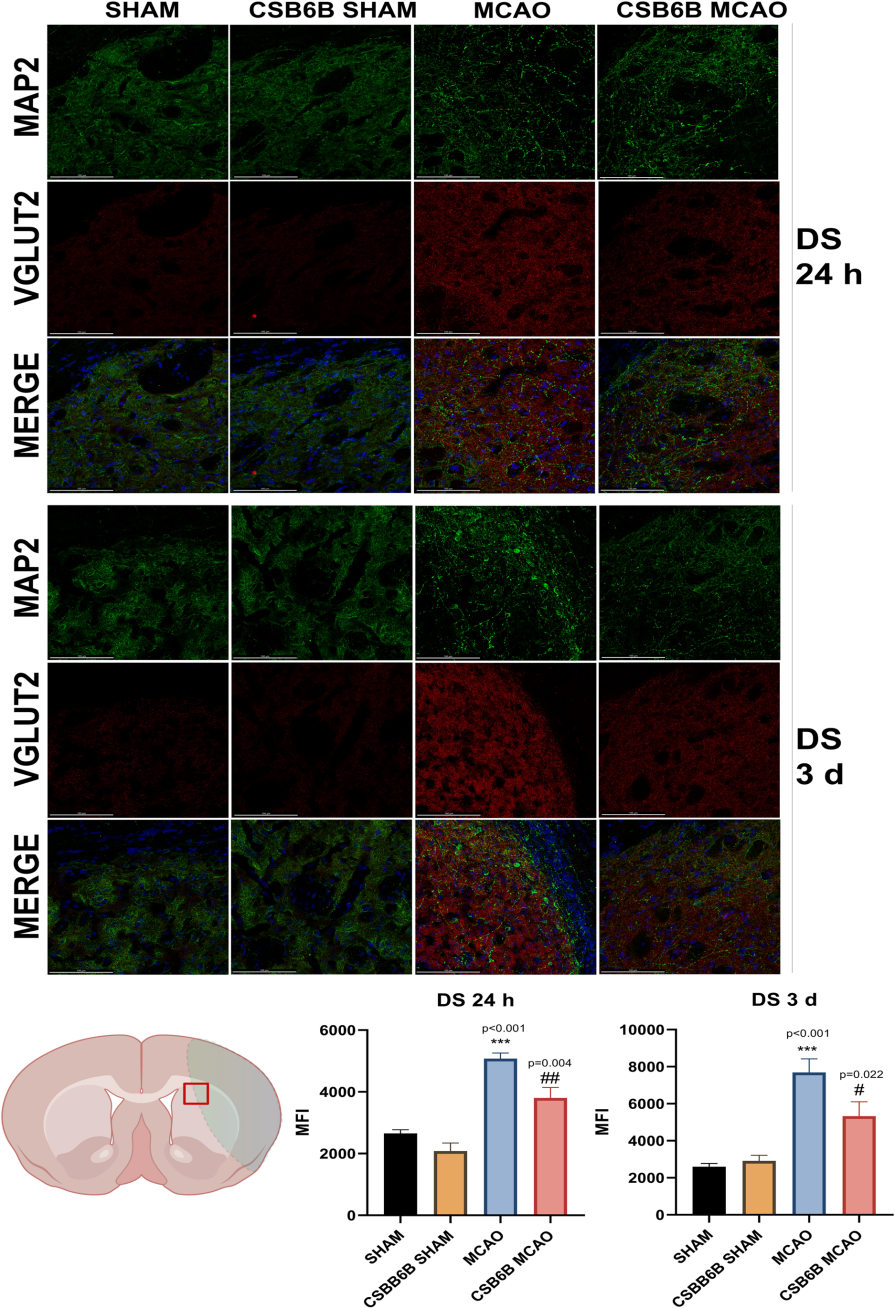
Expression of VGLUT2 in the dorsal striatum at 24 h and at 3 d after reperfusion. A total of 20 μm thick brain sections were double stained for MAP2 (green) and VGLUT2 (red). For each experimental group, *n* = 6 animals. Below on the left, on a brain diagram, the periinfarct area marked with red square indicates the location of performed analysis. Below on the right, the quantitative analysis of VGLUT2 mean fluorescence intensity (MFI). Data were analyzed with one-way ANOVA, followed by Sidak post hoc test (*p < 0.05, **p < 0.01, ***p < 0.001 for comparisons vs SHAM; #*p* < 0.05, ##*p* < 0.01 for comparisons vs MCAO). Scale bar = 100 µm

Moreover, we analyzed the alterations in VGLUT1 and VGLUT2 expression within the periinfarct area using double immunofluorescent staining of brain slices (Figs. 4, 5, 6, 7). In the frontal cortex of animals subjected to MCAO only, VGLUT1 was massively upregulated at both timepoints (24 h and 3 d), as revealed by fluorescence intensity analysis. An increased fluorescence intensity for VGLUT1 was also present in the dorsal striatum 3 d after reperfusion. CSB6B administration reduced the VGLUT1 signal in the frontal cortex of both experimental groups 24 h after surgery, but 3 d after reperfusion, we observed the reduction only in ischemic group. Pretreatment with CSB6B also reduced the immunoreactivity of the dorsal striatum in animals subjected to ischemia at 3 d after reperfusion. MCAO resulted in an increased fluorescence signal of VGLUT2 in the frontal cortex and in the dorsal striatum at 24 h and 3 d after reperfusion. CSB6B pretreatment decreased the VGLUT2 signal in the frontal cortex at 24 h, whereas in the dorsal striatum both, at 24 h and at 3 d after reperfusion.

### Pretreatment with CSB6B Reduces Glu Release in Brain Ischemia

**Fig. 8:**
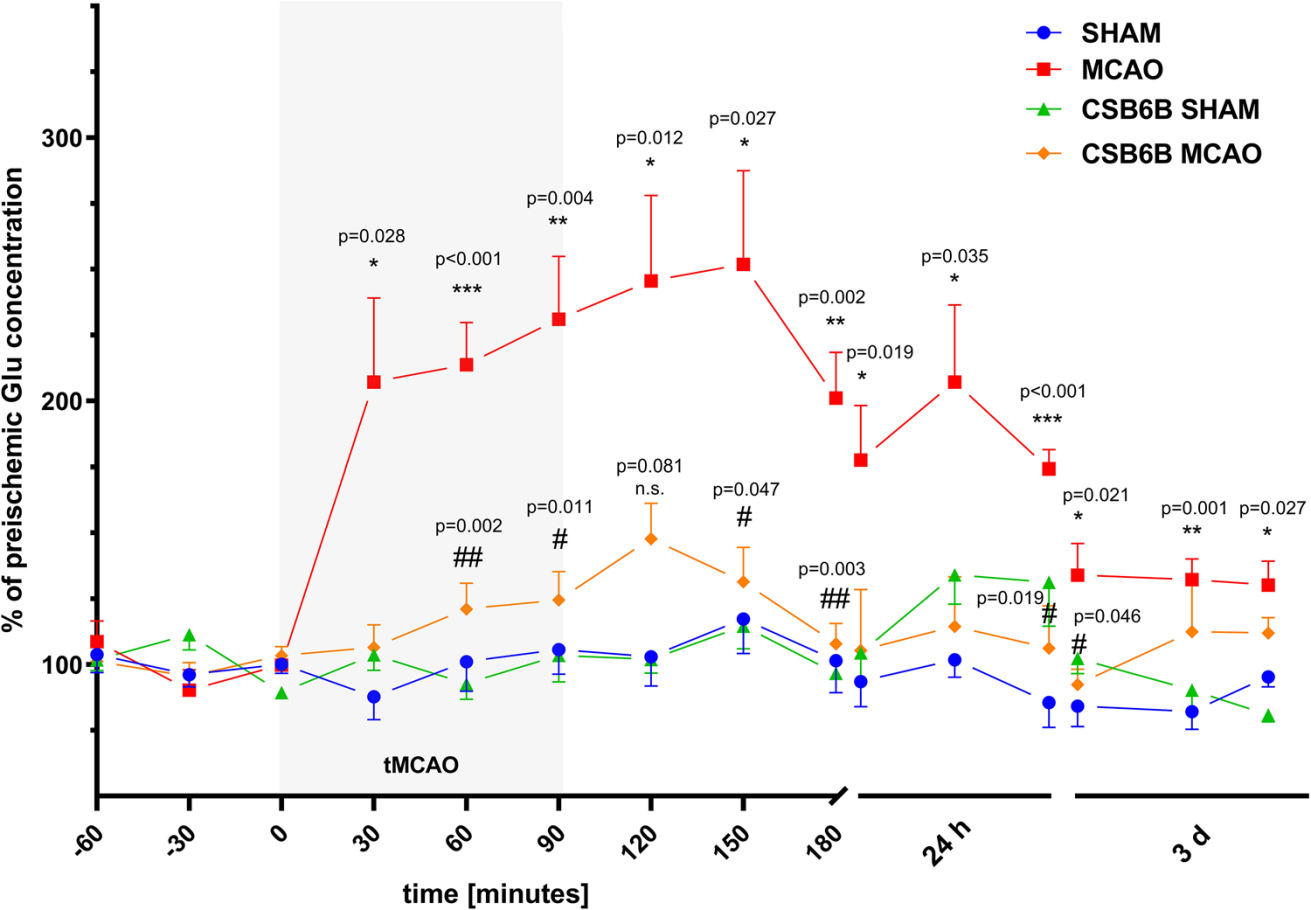
Extracellular Glu concentration changes in dialysate samples during ischemia/reperfusion in the cerebral cortex. Changes of Glu concentrations are expressed as % of preischemic Glu concentration. Data are presented as mean ± SEM (*n* = 8). Two-way repeated measures ANOVA was conducted, followed by Sidak post hoc test (*p < 0.05, **p < 0.01, ***p < 0.001 for comparisons vs SHAM; #*p* < 0.05, ##*p* < 0.01 for comparisons vs MCAO). Microdialysates were collected during the following periods: preischemia (− 60 to 0 min); ischemia (0–90 min), reperfusion (90–180 min), and next in the experimental setup involving freely-moving animals at three 30-min intervals 24 h and 3 days after reperfusion. CSB6B was administered intraventricularly at 2 h before MCAO surgery or SHAM procedure

MCAO induced a significant increase in Glu release into the brain parenchyma (Fig. 8). Elevated amounts of Glu were maintained during MCAO and up to 3 d after reperfusion. Administration of CSB6B prior to ischemia significantly reduced Glu release during artery occlusion and up to 90 min of reperfusion. Twenty-four hours after reperfusion, we observed that CSB6B pretreatment reduced the Glu concentration in the extracellular space compared to that in the MCAO group. Three days after MCAO, no considerable effects of CSBB6B administration on Glu concentration were present when compared to the MCAO group. Compared to control animals (SHAM), CSB6B alone did not significantly alter Glu levels at any timepoints or intervals (Fig. 8).

## Discussion

Glu loading into presynaptic vesicles is a critical step in Glu release into the extracellular space and subsequent consequences of Glu-related neurotransmission, both physiological and pathological [19]. In brain ischemia, immediately after its onset, there is a huge increase in extracellular Glu with an overstimulation of synaptic and extrasynaptic Glu receptors. This leads to calcium overload, mitochondrial disruption, energy deficit, transcription shout-down, and potential death of postsynaptic neurons. Glu-related excitotoxicity is a key component in the pathophysiology of brain ischemia. To date, all attempts at suppressing the damaging effects of excitotoxicity, including the inhibition of Glu receptors or activation of Glu uptake systems, have failed. VGLUTs are crucial for the first step of Glu transmission. Thus, their modulation may be beneficial in brain ischemia or other diseases related to Glu-related excitotoxicity. The existing data about the role of VGLUTs in brain ischemia are sparse and ambiguous. To the best of our knowledge, no reported studies have investigated the consequences of the pharmacological modulation of these transporters in focal cerebral ischemia. This could be related to the lack of specific and selective inhibitors of particular isoforms of VGLUTs. Azo dyes (including CSB6B) inhibit VGLUTs at nanomolar concentrations [20]; however, these compounds do not penetrate the blood‒brain barrier (BBB). Thus, to study the in vivo effects of VGLUT inhibition in ischemia‒reperfusion conditions, CSB6B was administered *i.c.v*.

The present study focused on the effects of transient focal cerebral ischemia on VGLUT1 and VGLUT2 expression in a spatiotemporal context. Furthermore, the consequences of VGLUT inhibition with CSB6B on Glu release and stroke outcome were investigated.

Brain ischemia‒reperfusion modulated both VGLUT1 and VGLUT2 expression (mRNA and protein) (Figs. 2 and 3). In the frontal cortex, 12 h and 3 d post-reperfusion, we observed a decrease in the VGLUT1 mRNA level, but interestingly, at 24 h, this level was increased. Similar changes were noticed for VGLUT2 mRNA in the frontal cortex; however, 12 h after reperfusion, the mRNA level was comparable with that in SHAM animals. Considering the temporal shift between the expression of mRNA and protein, RT‒PCR results are consistent with VGLUT1 and VGLUT2 protein data, i.e., increased levels of VGLUT1 and VGLUT2 proteins in the cortex at 3 d. The frontal cortex in the MCAO model is considered the periinfarct area where neurorepair mechanisms are activated. One may speculate that the upregulation of VGLUT1 and VGLUT2 expression later after ischemia results in increased Glu release and subsequent cortical excitability. It has been shown that increased extracellular levels of Glu in the cortex are related to an enhanced rate of neurogenesis within the periinfarct tissue [21–23]. Thus, the results of this study suggest that elevated expression of VGLUT1 and VGLUT2 in the acute phase (first 3 d) of cerebral ischemia may constitute a physiological neurorepair mechanism in the affected but still viable areas of the brain. The microdialysis results partially support this theory, since elevated extracellular Glu levels were maintained until 3 d post-reperfusion (Fig. 8). This could result from upregulated VGLUT expression, increased activity of these transporters, ineffective Glu uptake mechanisms, leakage of Glu from damaged cells, or, most likely, from all these processes together. The elevated excitability may stimulate neurogenesis, and there are studies showing noticeable neurogenesis in the rat brain 3 days after ischemia, with a peak at 7 days [24]. RT‒PCR results for 3 d suggest that this increased expression of both transporters in the cortex is transient. Since persistent neurogenesis may result in the formation of malfunctional neuronal connections [25], decreased cortical mRNA levels of VGLUT1 and VGLUT2 at 3 d might be a part of a physiological mechanism restoring basal functions in the periinfarct zone.

The dorsal striatum is the core of ischemia, and the cellular damage here is the most prominent. Twenty-four hours after reperfusion, a similar upregulation of VGLUT1 mRNA was present as in the cortex and was maintained up to 3 d. Following this raised VGLUT1 mRNA level, an increased protein presence was noticeable. These changes might be explained by similar cortical neurorepair mechanisms; however, due to more severe neuronal damage, they are more intense. With respect to the VGLUT2 mRNA level in the dorsal striatum, a decrease at 24 h followed by an increase at 3 d was observed. However, these results did not correspond fully to VGLUT2 protein expression, which was only increased at 24 h. A massive loss of neurons in the dorsal striatum, along with astrocyte activation, occurs after ischemia onset. Since it is thought that astrocytes express VGLUT2 [26], their activation might be a possible explanation for this data.

Our attempt to inhibit VGLUT activity before focal brain ischemia onset revealed a neuroprotective effect comparable in magnitude with that of ischemic preconditioning. Administration of CSB6B 90 min prior to MCAO significantly reduced the neurological deficit and infarct volume. Analyses of the effects of CSB6B administration prior to MCAO on VGLUT mRNA and protein expression revealed that CSB6B either maintained the expression of transporters at the basal (SHAM) level or caused a significant decrease in expression. At 12 h post-reperfusion, CSB6B did not change the mRNA expression of either transporter in the sham group, whereas in the ischemic group, a decrease in VGLUT2 was observed after CSB6B treatment only in the dorsal striatum, and the expression level in this brain area was maintained 24 h and 3 d after ischemia‒reperfusion. At 24 h after reperfusion, CSB6B reduced VGLUT1 mRNA levels in both brain structures and reduced VGLUT2 levels in the cerebral cortex, and these effects were also maintained at 3 d.

The protein levels of VGLUT1 and VGLUT2 in the cortex of animals administered CSB6B were markedly reduced 24 h after reperfusion in both experimental groups subjected to ischemia or sham procedures. The mechanisms underlying this downregulation remain, however, unclear. In the dorsal striatum at 24 h, CSB6B preserved the expression of VGLUT2 at the basal level (compared to the SHAM groups). Similarly, 3 d after ischemia, CSB6B maintained the expression of VGLUT1. Interestingly, CSB6B administration did not result in significant alterations in VGLUT1 and VGLUT2 protein expression in the cortex at 3 d. On the other hand, administration of CSB6B before MCAO greatly reduced the amount of Glu in the extracellular space. This reduction commenced just after ischemia onset and was evident up to 3 d after reperfusion. In animals receiving CSB6B but not subjected to MCAO, the extracellular level of Glu was comparable with that in the sham group, which may suggest that CSB6B administration does not disturb physiological glutamatergic transmission but prevents excessive release of Glu in response to brain ischemia.

Altogether, the results of this study suggest that compounds acting similarly to CSB6B may possess neuroprotective activity. First, long-term downregulation of VGLUT activity was observed, and second, reduced VGLUT expression was observed.

However, this study has some limitations. In addition to blocking VGLUTs, CSB6B is a potent inhibitor of macrophage migration factor (MIF) [27, 28]. Thus, CSB6B may possess an additional anti-inflammatory effect. In cerebral ischemia, neuroinflammation is to some extent coregulating both the neurotransmission as well as the cellular structure of the infarct and periinfarct site. Therefore, it is unclear whether the effect of CSB6B on the infarct volume or Glu level results from the modulation of VGLUT activity and expression or is an effect of MIF inhibition, or we may be observing an additive effect. Moreover, CSB6B was administered before ischemia onset. However, from the clinical point of view, the most important therapeutic strategy is the one that might be applied during the acute phase of stroke. This study aimed to determine the effect of MCAO on the expression of VGLUT1 and VGLUT2. Finally, CSB6B and other azo dyes are nonselective and nonspecific in their mode of action, and these compounds also do not cross the BBB. Therefore, to better understand the functions of VGLUTs as a potential therapeutic target and to fill the abovementioned knowledge gaps, better pharmacological tools need to be developed.

## Methods

### Animals and Experimental Design

All experiments were performed on male Sprague‒Dawley rats (280–320 g, Charles Rivers). Animals were randomly allocated into the following experimental and timepoint groups: the SHAM 12 h, MCAO 12 h, CSB6B SHAM 12 h, CSB6B MCAO 12 h, SHAM 24 h, MCAO 24 h, CSB6B SHAM 24 h, CSB6B MCAO 24 h, SHAM 3 d, MCAO 3 d, CSB6B SHAM 3 d, CSB6B MCAO 3 d. Each group had an n of 6–8 animals. Timepoints 12 h, 24 h, and 3 d refer to the time lapse between the onset of reperfusion and animal decapitation and tissue collection. The animals were maintained on a normal day-night cycle at 22 ± 2 °C with free access to food and water. The experimental protocols were in accordance with the Guide for the Care and Use of Laboratory Animals published by the National Institutes of Health and were approved by the First Local Ethical Committee at Jagiellonian University in Krakow (permit no: 11/2017). All studies involving animals are reported according to the ARRIVE (Animal Research: Reporting of In Vivo Experiments) guidelines, including the procedure for blinding the investigators to the identities of the animals at each point of the experiment. CSB6B or artificial cerebrospinal fluid as a control was administered to rats 2 h before MCAO. CSB6B (1 mg/mL, dissolved in artificial cerebrospinal fluid) was slowly injected into the contralateral ventricle (*i.c.v.*) (anteroposterior (AP): 0.0 mm; mediolateral (ML): 1.6 mm; dorsoventral (DV): 4.0 mm, all coordinates according to the bregma) at a volume of 5 μL (0.5 μL/min) using a stereotaxic frame. The dosage was based on our preliminary studies and available literature [29]. The efficacy of administration was confirmed during brain tissue collection.

### Focal Cerebral Ischemia Model

MCAO was elicited according to the method of Longa et al. as previously described [30]. All surgical procedures were carried out under a stereoscopic microscope (Leica, A60F; Germany), and body temperature was maintained at a physiological level using a heating blanket (Homeothermic Blanket System; Harvard Apparatus). Arterial occlusion was confirmed using the laser speckle contrast analysis system (PeriCam HR PSI, Perimed, Sweden), and a 70% blood flow reduction was considered to indicate a successful procedure. The rats were anesthetized with 5% isoflurane for induction and 2.5% isoflurane for maintenance. After exposure of the left external carotid artery (ECA), the internal carotid artery (ICA), and the common carotid artery (CCA), all branches of the ECA were coagulated. The ECA, ICA, and CCA were temporarily secured with microvascular clips. A silicone-coated filament (Doccol, USA) was introduced into the lumen of the ECA and advanced until the blood flow decreased. The clip was removed from the CCA, and the wound was secured with silk sutures. The occlusion was maintained for 90 min. Afterward, the wound was reopened, the filament removed to restore blood flow, and the wound closed. The sham operation was carried out as described above without insertion of the filament. The mortality rate for MCAO was 4% for 12 h timepoint, 6.2% for 24 h timepoint, and 9.35% for 3 d timepoint. The success rate of the optimal MCA occlusion was 86.4%.

### Neurological Deficit

Neurological deficits were assessed at 12 h, 24 h, and 3 d after reperfusion. For each assessed group, *n* = 8 animals. The 10-point grading system of Philips et al. (2000) was used [31], as previously described [32]. The following neurological symptoms were assessed and scored: 4 points were given when animals pushed in a contralateral direction did not show any resistance, 3 points were given when animals circled in a contralateral direction, if animal demonstrated contralateral shoulder adduction, 2 points were added, and 1 point was given if contralateral forelimb flexion was observed. Zero indicates a lack of neurological deficit, whereas 10 points was the maximal neurological deficit.

### TTC Staining and Measurement of the Infarct Volume

To determine the infarct volume, separate experimental groups were prepared (for each group, *n* = 6). Twenty-four hours after MCAO, animals were decapitated, and their brains were immediately removed and cut using a brain matrix (Harvard Apparatus, USA). Coronal Sects. (2 mm thick) were stained with a 1% solution of 2,3,5-triphenyltetrazolium chloride (TTC) dye (Sigma Aldrich, USA) in 0.01 M phosphate-buffered saline (Sigma Aldrich, USA) at 37 °C for 10 min in the dark. Next, sections were fixed in 10% phosphate-buffered formalin (Sigma Aldrich, USA) for 30 min at 4 °C. The stained and fixed brain slices were photographed using a surgical microscope equipped with a camera (Leica S9D with Flexacam C3, Leica, Germany) by an investigator blinded to the subject identity. The infarct volume was determined using NIH ImageJ software (National Institutes of Health, version 8.0) by the same investigator. The infarct volume was calculated as a sum of each outlined white area multiplied by the thickness of the brain section and was expressed in mm^3^.

### Microdialysis of the Motor Cortex

Guide cannulas were implanted 24 h prior to microdialysis. The animals were anesthetized with 2.5% isoflurane and then stereotaxically implanted with guide cannulas (MAB 4; AgnTho’s, Sweden) aimed at the frontal cortex (anteroposterior (AP): − 0.48 mm; mediolateral (ML): + 2.0 mm; dorsoventral (DV): − 1.2 mm) according to the atlas of Paxinos and Watson (2007). The guide cannulas were affixed to the skulls with acrylic dental cement and cranial screws. Obturators were placed in the cannulas until the microdialysis probes were inserted. Next, the obturators were removed from the guide cannulas, and microdialysis probes (MAB 4, membrane with a molecular weight 6-kDa cut off, 2-mm length, 0.24-mm outer diameter, AgnTho’s AB, Sweden) were inserted into the guide cannulas. To wash inserted probes, brain structures were perfused for 2 h with artificial cerebrospinal fluid (aCSF) (in mM: 147 NaCl, 4.0 KCl, 1.0 MgCl_2_, 2.2 CaCl_2_, pH 7.4) at a constant flow rate (2 μL/min). After this period, samples were collected every 30 min as follows: two baseline samples, three samples during MCAO, and two samples during the reperfusion period. At 24 h and at 3 d after reperfusion, the microdialysis procedure on freely-moving animals was repeated, maintaining 2 h washing period of the probe, followed by three samples collection every 30 min. All samples were immediately frozen and stored at − 80 °C until the LC‒MS assay. For each experimental group, *n* = 8 animals. After collecting the last sample at 3 d after reperfusion, the localization of guide cannula in the motor cortex was verified.

### LC‒MS Analysis of Glu

The chromatographic separation was performed on a Waters ACQUITY UPLC® H-Class system (Waters Corporation, Milford, MA, USA) comprised of a quaternary pump, column oven, autosampler (with 100 µL of ANSI-384 well), and photodiode array detector. The MS/MS instrument was a Xevo TQ-S® mass spectrometer operating in positive electrospray ionization mode, and two multiple reaction monitoring (MRM) transitions were monitored per component. Optimization of the MS/MS parameters was performed by infusion of 1 µg/mL standard solutions of each analyte and IS into the Xevo TQ-S® mass spectrometer at a flow rate of 10 µL/min, in combination with the mobile phase (50% A/50% B, flow rate: 0.2 mL/min) using the IntelliStart Fluidics system.

The aCSF samples were analyzed in gradient mode with a ZIC®-HILIC column (5 µm, 200 Å, 150 × 21.2 mm; Merck, Darmstadt, Germany). The temperature of the column thermostat was set at 40 °C. The flow rate of the mobile phase was 0.3 mL/min. The mobile phase consisted of water with the addition of 0.02 M formic acid (solvent A) and acetonitrile with the addition of 0.02 M formic acid (solvent B). The initial gradient of solvent A was 50% (for 0.06 min), then was decreased to 3% (for 0.6 min), maintained at 3% (for 1.2 min), and finally increased to 50% (for last 2.8 min.). Pairs of ions were monitored in the assay using the following values of m/z: 148.1/84.1 for Glu and 153.1/89.1 for Glu-C13 (Internal Standard). The results were analyzed using MassLynx software V4.2 (Waters, Milford, MA, USA). Levels of Glu were calculated using the calibration standard curves, which were constructed by linear regression analysis of peak area versus concentration.

The ion source parameters were as follows: ion spray voltage (IS): 5400 V; nebulizer gas (gas 1): 30 psi; turbo gas (gas 2): 20 psi; temperature of the heated nebulizer (TEM): 550 °C; curtain gas (CUR): 30 psi. Nitrogen (99.9%) from Peak NM20ZA was used as the curtain and collision gas. The quantitation analysis was performed using the MRM mode and tandem LC/MS.

### Western Blot

The tissues of the frontal cortex and dorsal striatum were homogenized in 2% SDS containing 1 mM PMSF, 1 mM Na_2_VO_4_, 20 mM NaF, and a mixture of phosphatase-proteinase inhibitors (Sigma Aldrich) using Ultra-Turrax and ultrasonic homogenizers. After denaturation at 95 °C for 10 min, insoluble debris was removed by centrifugation at 10,000 × g for 10 min at 4 °C. The protein concentration in the supernatants was determined using a BCA protein assay kit (Thermo Scientific, USA). After setting a proper protein concentration, the solutions were mixed with loading buffer (containing 10% 2-mercaptoethanol) at a ratio of 1:1 and heated for 10 min at 95 °C. Samples were loaded on gradient 8–16% SDS polyacrylamide gels (Criterion, TGX-Stain-free gel, Bio-Rad) at a total protein concentration of 30 µg/10 µL, and electrophoresis (200 V, 45 min) was performed. Next, proteins were semidry transferred (TurboBlot, Bio-Rad) to PVDF membranes, and the total protein content on the membrane was visualized with stain-free procedure. Next, membranes were blocked in a 5% solution of albumin. Membranes were incubated overnight at 4 °C with primary antibodies at the appropriate concentration. After an overnight incubation, the membranes were washed in TBST and then in 1% albumin solution and incubated with the respective secondary antibodies conjugated with peroxidase in 1% albumin solution for 1 h at RT. After washing, the membranes were developed using the ECL method (Western Bright Quantum, Advansta Inc., USA). The chemiluminescence of the membranes was imaged with a G-Box Imaging System (Syngene, USA), and the protein expression was analyzed with Gene Tools software (Syngene, USA) and expressed relative to the total protein content in the sample. Each experimental group consisted of *n* = 6 animals.

### RT‒qPCR

The rats were decapitated 12 h, 24 h, or 3 d after reperfusion or sham operation. The brains were removed, and the ipsilateral brain structures were isolated and immersed in *fixRNA* solution (EURx, Poland) for 24 h at 4 °C to preserve RNA. Next, total RNA was extracted using TRIzol reagent and purified with a microcolumn system according to the manufacturer’s protocol. The concentration and purity of the RNA were determined by measuring the A260/A280 ratio with a Nanoquant plate (Tecan, Austria). cDNA was synthesized using 2 µg of total RNA and a Smart First Strand cDNA Synthesis Kit (EURx, Poland) according to the manufacturer’s protocol. The cDNA was stored at − 80 °C until use. RT‒qPCR amplification was performed on a CFX Connect system (Bio-Rad, USA) using appropriate TaqMan Gene Expression Assays (Applied Biosystems, USA), 200 ng of template cDNA and Probe qPCR Master Mix (EURx, Poland) according to the manufacturer’s instructions. The thermal cycling conditions were as follows: 95 °C for 15 min (initial denaturation) followed by 35 cycles of 94 °C for 15 s (denaturation), 55 °C for 30 s (annealing), and 72 °C for 30 s (extension). Reactions for each sample were performed in triplicate. The efficiency of the PCR for each TaqMan probe was verified. The fold change in the expression of each gene was calculated using the ΔCt method using three housekeeping genes, *Ppia*, *Ywhaz*, and *Hprt*, as we described recently [33]. Next, these values were used for further statistical analysis. The TaqMan probes used for RT‒qPCR are listed in Table 1. Each experimental group consisted of *n* = 6 animals.

**Table 1:** Characteristics of TaqMan™ probes

Gene symbol	Assay ID	Assay design	Amplicon length
*Ppia*	Rn00690933_m1	Probe spans exons	149
*Ywhaz*	Rn00755072_m1	Probe spans exons	104
*Hprt*	Rn01527840_m1	Probe spans exons	64
*Vglut1* (*Slc17a7*)	Rn01462431_m1	Probe spans exons	81
*Vglut2* (*Slc17a6*)	Rn00584780_m1	Probe spans exons	141

### Tissue Fixation for Immunofluorescent Double Staining

Groups of animals (*n* = 6 animals per group) used for immunofluorescent staining were subjected to intracardiac 4% paraformaldehyde (PFA) perfusion 24 h after the surgical procedure. First, the animals were deeply anesthetized with ketamine (80 mg/kg) and xylazine (20 mg/kg). Then, the rats were transcardially perfused with 250 mL of NaCl solution (0.9%; 32 °C) until all the remaining blood was removed. Next, animals were perfused with 500 mL of 4% PFA in a 0.1 M PBS. After perfusion, the brains were removed and kept in the same 4% PFA solution overnight. Afterward, the brains were transferred to 10% sucrose solution containing 0.1% sodium azide in 0.1 M PBS and stored for 24 h. Next, the brains were transferred to 20% and then to 30% sucrose solution with 0.1% sodium azide in 0.1 M PBS and stored until they sank. The brains were then cut into 20 μm coronal sections using an automatic cryotome (Leica CM 1860). The sections were mounted on SuperFrost (Thermo, USA) microscopic slides and stored at − 20 °C until staining and analysis.

### Immunofluorescent Double Staining

Brain sections were placed on microscopic slides and stored at − 20 °C until staining. A double immunostaining procedure was performed to visualize the colocalization of either VGLUT1 or VGLUT2 proteins with the neuronal marker MAP2; thus, appropriate mixtures of the two antibodies were used for tissue staining. The antibodies used in this method are listed in Table 2. First, slides were immersed in 70% ethanol for a few seconds and air dried for 3 min. Slides were washed twice in 0.01 M PBS for 10 min. Next, the slides were washed once in 0.3% Triton X-100 in 0.01 M PBS for 10 min in order to permeabilize. Nonspecific binding was blocked by incubating slides in 10% goat normal serum (GNS) in 0.3% Triton X-100 in 0.01 M PBS for 1 h. Next, tissues were incubated in primary antibody solutions overnight at 4 °C in humidity chambers. Primary and secondary antibody solutions were prepared by in 2% GNS in 0.3% Triton X-100 in 0.01 M PBS. The following day, the slides were washed twice in 0.3% Triton X-100 in 0.01 M PBS and then twice in 2% GNS in 0.3% Triton X-100 in 0.01 M PBS. Each of these washes was for 10 min. Tissues were incubated in secondary antibody solution for an hour in the dark. Next, the slides were washed four times in 0.01 M PBS for 10 min. All washing and incubation steps were performed using a rocking shaker. Slides were dried with a paper towel, covered in mounting medium (Vectashield Vibrance with DAPI, Vectorlabs) and coverslipped. Slides were stored at 4 °C until visualization. Images were obtained using a Leica Stellaris 8 WLL DLS confocal microscope and Leica LAS X software. Each scan of the particular staining of the particular brain structure was performed using the same settings of the microscope, including the same pinhole (0.9 AU), the same power of lasers and detector, the same laser frequency (300 Hz) as well as the same characteristics of the z-stack and similar part of the brain structure located on the border between the core of ischemia, and the periinfarct zone. The resolutions of gathered scans were 2048 × 2048 pixels. The fluorescence intensity was analyzed in LAS X software selectively for each focal plane, before stitching the z-stack (35 focal planes). The fluorescence intensity analysis refers to the total surface of brain slice visible in a scan of a singular focal plane, corrected by the fluorescence of the background. Results are expressed as a mean fluorescence intensity in mean fluorescence intensity (MFI); for each experimental group, *n* = 6 animals; for each animal brain slices were scanned at 3–4 positions per brain structure (cortex or dorsal striatum), each position is a z-stack consisting 35 scans of focal planes.

**Table 2:** The list of antibodies used in immunofluorescent double staining protocol

Antibody	Dilution	Host	Cat #	Supplier
Anti-VGLUT1	1:1000	Mouse	AMAb91041	Sigma Aldrich
Anti-VGLUT2	1:200	Mouse	AMAb91081	Sigma Aldrich
Anti-MAP2	1:750	Chicken	PA1-10,005	Invitrogen
Goat anti-mouse Alexa Fluor Plus 488	1:400	Goat	A32723	Invitrogen
Goat anti-chicken Alexa Fluor Plus 594	1:400	Goat	A32759	Invitrogen

### Statistical Analyses

All data are expressed as the mean ± standard error of the mean (SEM). The infarction volume and protein and mRNA expression data were analyzed using one-way ANOVA. If statistical significance was found after ANOVA, Sidak’s post hoc test was conducted to test the comparisons between experimental groups. Microdialysis results were analyzed by two-way repeated-measures ANOVA with the Sidak post hoc test. A Mann‒Whitney *U* test was used for the analysis of neurological deficits. Each statistical analysis compared the following groups: treatment alone, treatment + MCAO, sham, and MCAO. A calculated *p* value < 0.05 was considered statistically significant. Calculations were performed using GraphPad Prism software ver. 8.2.1 (GraphPad Software, USA).

## Data Availability

Data will be made available on reasonable request.
